# Winnow based identification of potent hERG inhibitors *in silico*: comparative assessment on different datasets

**DOI:** 10.1186/1758-2946-4-S1-O6

**Published:** 2012-05-01

**Authors:** Richard L Marchese Robinson, Robert C Glen, John BO Mitchell

**Affiliations:** 1 Unilever Centre for Molecular Science Informatics, Department of Chemistry, University of Cambridge, Lensfield Road, Cambridge, CB2 1EW, UK; 2 EaStCHEM School of Chemistry and Biomedical Sciences Research Centre, University of St Andrews, North Haugh, St Andrews, KY16 9ST,UK

## 

Due to the potentially lethal effects of potent hERG inhibition, *in silico* approaches which can identify potent (IC_50_ < 1 µM) inhibitors are of considerable interest to the pharmaceutical industry [1]. We present recent work [2] in which *in silico* binary classifiers were trained to discriminate potent inhibitors from compounds exhibiting weaker (IC_50_ ≥ 1 µM) inhibition. Initial models were based on a version of the memory efficient Winnow algorithm [3]. These initial models were generated using various descriptor sets. The descriptor set yielding the best cross-validated initial Winnow model was used to build models using each of Winnow, Random Forest and Support Vector Machine. Analysis of the contributions of different substructural and physiochemical features in the final Winnow models indicates they may be interpreted, albeit with caution. All final models were externally validated, with no algorithm consistently outperforming the others. These approaches were directly compared, on various datasets, to those proposed by Thai and Ecker [4] and by Dubus et al. [5]. The results indicate that the Winnow models are competitive with earlier approaches proposed in the literature. The findings also emphasise a potential difficulty when seeking to estimate the predictive power of *in silico* models on small quantities of data: model performance may vary considerably, particularly when training and validating on different datasets.
